# Progress in improving provincial plans for nutrition through targeted technical assistance and local advocacy in Vietnam

**DOI:** 10.1093/heapol/czw067

**Published:** 2016-05-19

**Authors:** Jody Harris, Phuong H. Nguyen, Quyen To, Edward A. Frongillo, Purnima Menon

**Affiliations:** ^1^Poverty, Health and Nutrition Division, International Food Policy Research Institute, Washington, DC, USA; ^2^Arnold School of Public Health, University of South Carolina, Columbia, SC, USA; ^3^FHI360 Vietnam, Hanoi, Vietnam

**Keywords:** Nutrition, planning, decentralization, Vietnam

## Abstract

Vietnam has been decentralizing nutrition planning to provinces, which could help with local relevance and accountability. Assessment in 2009 found a continuing top-down approach, limited human capacity, and difficulty in integrating multiple sectors. Alive and Thrive (A&T) provided targeted assistance and capacity-building for 15 provincial plans for nutrition (PPNs). We aimed to (i) assess PPN content and quality improvements 2009–2014, and (ii) explain processes through which change occurred. Data consisted of interview-based assessments of provincial planning processes, annual PPN assessments, and tracking of A&T involvement. At endline, some provinces produced higher quality plans. Local planning skills improved, but capacity remained insufficient. Awareness of and support for nutrition improved, but some policy and legal environments were contradictory. Objectives were clearer, but use of data for planning remained inconsistent. Provinces became more proactive and creative, but remained constrained by slow approval processes and insufficient funding. Targeted assistance and local advocacy can improve decentralized planning, with success dependent on policy and programming contexts and ability to overcome constraints around capacity, investment, data use and remnants of centralized planning. We recommend strong engagement with planners at the national level to understand how to unblock major constraints; solutions must take into consideration the particular political, financial and administrative context.

Key Messages

- Targeted assistance and local advocacy can improve decentralized planning.
- Success is dependent on policy and programming contexts and ability to overcome constraints around capacity, investment and data use.
- A key issue is the degree to which centralized planning can be devolved; strong engagement is needed with planners at national level to achieve this.
- Solutions must understand and consider the particular political, financial and administrative context in which decentralized planning is being attempted.

## Introduction

A broad theme of public administration in general, and of nutrition for development in particular, is that centralization of power and resources with national bodies limits program applicability and accountability locally (Bryce *et al.* 2008). The solution proposed in an increasing number of states is varying forms of decentralization, transferring different types of power, to different extents, to different forms of local authorities (Ahmad *et al.* 2005). Decentralization theory holds that service quality can be improved through many and diverse routes, including engagement of local citizens in decisions (legitimacy); holding local leaders to account and reducing corruption (integrity and accountability); tailoring of services and use of resources to local contexts (allocative efficiency); linking local revenues to services (cost recovery); improvement of local capacity (technical efficiency); service delivery innovation (quality); fairer distribution of resources (equity); and avoidance of delays due to central decision making (Litvak *et al.* 1998; Azfar *et al.* 1999, 2001; Bossert and Beauvais 2002). Empirical work has not uniformly upheld the theory; however, noting failures of policy coordination and failures due to inadequate capacity (Azfar *et al.* 2001); widening of regional disparities and short-term service disruption (Azfar *et al.* 1999); and elite capture of local government and weak accountability (Crook 2003) as outcomes of decentralization. This discrepancy between empirical observations and what is posited by theory may stem from imperfect theory or incomplete implementation or both, but which is the likely explanation is little understood. Given the possible positive and negative effects, the question for some becomes not whether to decentralize, but how to do it best for a given issue in a given context (Bossert and Beauvais 2002).

‘Decentralization’ refers to a range of localized governance arrangements which transfer power from a central authority to lower hierarchical levels, including ‘devolution’ (shifting decision-making powers to lower levels of government), ‘deconcentration’ (shifting responsibility or tasks but not decision-making power to geographically dispersed agents), and ‘delegation’ (shifting responsibility to entities outside of full government control) (Bossert and Beauvais 2002); these terms are not used consistently (Yuliani 2004). Decentralization takes three forms: administrative decentralization, where decisions and authority over planning and implementation of programs are delegated downwards; fiscal decentralization, where the means to accumulate and/or spend public money are afforded to local administrations; and political decentralization, whereby political choices are also made at local levels (Riedl and Dickovick 2014).

Decentralization is often viewed by states as a loss of power, both relinquishing central control and delegating power to sub-national elements which do not necessarily support the central government (Gilson *et al.* 2006; Maluka *et al.* 2011). Some states tend to choose decentralization as an administrative tool, and authoritarian, hegemonic regimes without political alternation—particularly those with strong sub-national footholds, a history of patronage of loyal local leaders, and high territorial penetration of functionaries with administrative capacity—are most likely to decentralize (Riedl and Dickovick 2014). In Vietnam, attempts to decentralize started in the late 1980s after the adoption of renovation (*doi moi*) (Nguyen 2008); the government has in recent years added decentralization of nutrition planning to provinces in an attempt to improve local relevance and accountability, in line with decentralization theory. In general, Vietnamese policy statements support administrative and fiscal decentralization to improve the effectiveness and transparency of the government (Fritzen 2006). Vietnam’s decentralization, however, faces two important challenges. First, bureaucratic actors lack incentive to transfer power and resources to local levels. Second, decentralization contributes to widening regional disparities due to differences in capacities and resources of local governments in rich and poor provinces (Fritzen 2006). Vietnam’s decentralization of public services has been described as ‘deconcentration’ rather than ‘devolution’, with the transfer of responsibility for specific tasks and associated funds, but maintenance of centralized decision-making power, targets, and plans (Wit 2007). Planning for nutrition presents several challenges that relate to the overall process of decentralization in Vietnam (Lapping *et al.*, 2014), the most serious among them being planning that is top down and financially directed rather than localized and having a problem-solving focus, as well as limited actor capacity for planning.

Reducing undernutrition lends itself to decentralized planning because of its intersectoral and context-specific nature. The convergence of multiple sectors and programs necessary for improved nutrition may be more feasible at local levels, and local combinations of causes may be easier to identify than when planned centrally (Harris and Drimie 2012). The Vietnamese government has had a particular interest in human capital in its social development strategy, and the National Nutrition Strategy (NNS) is seen as an inseparable part of the economic and social development strategy of the country. To implement the NNS on a nationwide scale, the government issued a decentralization policy to support the provinces in developing more effective plans for health and nutrition and respond appropriately to the needs of their own communities. Because most national programs for poverty alleviation and health promotion in Vietnam are managed by provinces (Green and Dao 2005), the provincial level is considered best to enact change in these issues (Lapping *et al.* 2007). Thus, improvements in provincial planning have potential for significant nutrition program impacts in theory, increasing local relevance, and the effectiveness of local plans. A nutrition plan that is well-justified, evidence-based, contextual and involves inter-sectoral stakeholders is also more likely to be funded by multiple sponsors, and such a plan can also lead to more efficient use of resources and ensure that these resources are used to better meet local needs, in theory resulting in narrowing disparities between rich and poor provinces.

In 2010 research was conducted to gain better understanding of the process of developing provincial plans for nutrition (PPNs) from different stakeholders in this process. The process of PPN development, review and approval annually was found to be lengthy, starting with activities undertaken at the national level, with implementation of tasks at the lower levels directed by authorities at the higher levels. Research conducted in seven provinces between 2009 and 2010 (Lapping *et al.* 2014) found that the effectiveness of PPN development was constrained by a top-down planning approach, limited budget, limited data availability and quality, limited staff experience or knowledge about process and finances for effective planning at sub-national levels, and difficulty in integrating actions from multiple sectors. Analysis of the PPN process at the provincial level suggested that it was largely driven by fiscal concerns, with little variation across administrative units; that is, the administrative units obeyed their (centrally provided) instructions to be best of their ability, based on prevailing resource and capacity constraints. Additional constraints identified—top-down processes and difficulty integrating sectors—were in large part due to broader issues related to decentralization of power. Authors concluded that transforming provincial planning from a top-down approach would be slow, with a strategic multiyear approach needed to strengthen the planning process in provinces, and requiring addressing key barriers identified through the research.

A consortium of implementers from *Alive and Thrive* (A&T), knowing about and partly responding to these findings, provided targeted assistance and capacity-building in 15 provinces of Vietnam over the period 2009–14 in order to improve nutrition planning. Health services research has found several conditions to be critical for success in decentralizing health-services planning in developing countries, including provision of adequate and appropriate assistance to local entities, formulating clear goals, carefully defining boundaries between functions controlled by central-level managers and those controlled by their field-level counterparts, and helping to build local-level capacity by providing technical and material support to field staff (Omar 2002). These functions were taken up in the A&T program (described in Box 1).

Box 1 Brief description of A&T and the goals of A&T’s work with respect to provincial planning

Alive & Thrive (A&T) is an initiative funded by the Bill and Melinda Gates Foundation to reduce under nutrition and death caused by sub-optimal and young child feeding practices in three countries (Bangladesh, Ethiopia and Vietnam) for a 6-year period (2009–14) (Baker *et al.* 2013). In Vietnam, A&T’s behavior change framework included four interrelated components: advocacy, interpersonal communication and community mobilization, mass communication, and strategic use of data. A&T Vietnam has been engaged in advocacy to improve the IYCF policy and regulatory environments for breastfeeding and to strengthen provincial planning on nutrition.

At the central level, A&T worked closely with the National Institute of Nutrition (NIN) and the Department of Maternal and Child Health Care to revise and develop the complex PPN preparation guidelines. A&T also supported NIN to prepare annual provincial nutrition profiles which contain critical nutrition data that can be used for evidence-based planning, and supported NIN financially (i.e. funding to organize provincial workshops) and technically (i.e. preparing presentations and providing technical support at workshops) in hosting PPN workshops. In addition, two national workshops were organized in 2010 and three in 2011 to review the implementation of the NNS 2001–10, disseminate strategies for 2011–15, guide the development of the PPNs, and inform the budget for each province.

At the provincial level, the main advocacy activities included information gathering, various types of capacity building and dissemination workshops, and hands-on support for preparing the PPNs. A&T staff worked closely with provincial partners to provide technical support in planning workshops, such as helping with preparing agendas, presentations, and other related materials. A&T staff was also closely involved in gathering evidence and information for the preparation of PPNs.

The objectives of this study were to: (i) assess the changes in provincial planning on nutrition, including PPN content and quality improvements 2009–14, in the provinces that received A&T support; (ii) explain processes through which change to PPNs occurred, or failed to occur; and (iii) assess the contribution of A&T support to the changes in provincial planning on nutrition.

## Methods

Data consisted of stakeholder interviews in 2010 and 2014, annual PPN quality assessments from 2010 to 2014, and tracking of A&T involvement from 2010 to 2014. These three data sources were brought together in a narrative synthesis for final analysis. The study received Institutional Review

Board approval from the authors institutes. Each of the participating provinces gave verbal consent to use the PPNs for content analysis. Verbal consent for participating and having the interviews tape-recorded were obtained for all interview participants, and the endline interview research protocol was reviewed and approved by the authors’ institutes.

### Stakeholder interviews

Stakeholder interviews covered participants from different sectors with roles in PPN such as development of nutrition plans, reviewing and/or approving and implementation. Interviews focused on what changed in provincial planning for nutrition, why changes happened, how A&T are perceived to have contributed to change, barriers to change towards evidence-based planning, and ideas on improving the effectiveness of PPN development and implementation. Seven of 15 provinces that received support from A&T were selected for interviews, representing the three major geographic regions in Vietnam (North, Central and South) and different situations for infant and young child feeding (IYCF). For instance, early initiation of breastfeeding is 33% in the North, 57% in the Central and 30% in the South regions. Exclusive breastfeeding is 15% in the North, 38% in the Central and 7% in the South regions. Minimum dietary diversity is 55% in the North and 37–40% in the Central and South regions (MICS 2011). Forty-one interviews with a total of 66 participants were conducted, including 26 interviews with an individual respondent and 15 in-depth interviews with 2 or more participants; group interviews were done when an invited participant requested that her or his staff members, who were involved in nutrition planning, attend without prior notice to the research team. Participants came from organizations in different sectors, each playing a role in different steps of the PPN process; more than three quarters of the participants were from health organizations.

NVivo 10 software was used to organize, code, and analyze all interview data. An *a priori* approach was used to build the code list, in which the codes and themes were established prior to data analysis based on the content and structure of the interview guide. During the coding process, researchers wrote memos to record any emerging patterns, key issues, recurrent themes and concepts, allowing expanding and accommodation of emerging themes and sub-themes. Through reflection and interpretation, data-derived findings of common themes and patterns or relationships between categories and their properties were summarized. Frequency of reference by respondents was used to identify priority issues; contradictory perspectives, where they were found, were also highlighted in reporting and discussing each theme.

### Annual PPN quality assessments

To monitor changes in the quality of PPNs, analyses were conducted annually to examine the elements of PPNs and the quality of those elements in provinces supported by A&T, and to assess changes in the quality of PPNs from 2010 to 2014^1^. A qualitative content analysis was employed to assess the quality of PPNs of fifteen Vietnamese provinces including four Northern, five Central,and six Southern provinces. The coding scheme, adapted from the coding concepts, is a combination of a priori coding and emergent coding (Stemler 2001). The coding scheme covered the main elements of PPNs including their process, structure, goals and objectives, actions, evidence-based planning, and funding. The PPNs were examined along seven ‘dimensions’: objectives, multi-sector integration, contextual data, evidence-based planning, resource mobilization, feasibility and creativity. The ‘objectives’ of the PPNs were assessed using SMART criteria to determine whether they were specific, measurable, attainable, realistic and time-based.

Several strategies were used to ensure the reliability and validity of the analysis. Researchers involved in the coding scheme had been working closely in Vietnam so that all meanings of rating and categorizations were clarified. Two researchers independently applied the coding to ensure reliability. The themes of the coding scheme were validated by independent researchers and government officers responsible for reviewing PPN annually.

### Tracking of A&T involvement

Events and activities undertaken by A&T over time were compiled into an events database, using A&T documents and reports to summarize activities into a template in Excel. The template, which contained information about policy and advocacy events or strategies, was documented monthly for each year. For this study, activities and strategies relating to the provincial level were extracted from the database and summarized.

## Results

### Changes in content of plans

**Figure 1.**
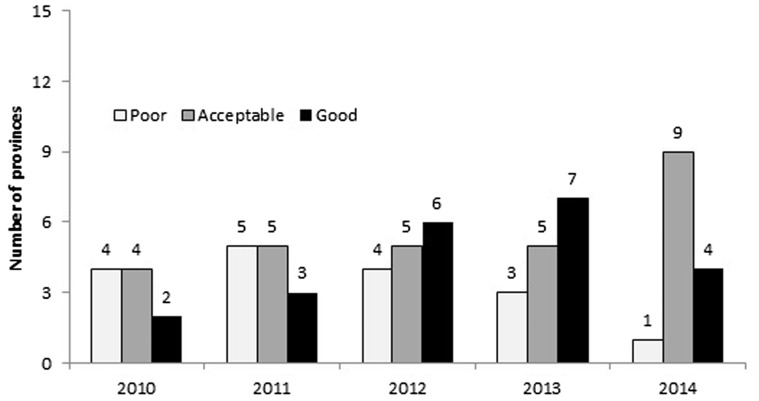
Overall assessment of PPNs from 2010 to 2014

Content of PPNs was assessed each year from 2010 to 2014 in each of the 15 provinces targeted by A&T. Marked improvements were seen over time in some elements of the plans, but stagnation in others. For the overall assessment assigned to the content of PPNs from 2010 to 2014, more plans were rated ‘good’ or ‘acceptable’ over time, though fewer were rated ‘good’ in 2014 than the previous year (Figure 1). There was only one province with a ‘poor’ overall assessment in 2014 compared to four in 2010. In general, the overall quality of the PPNs improved over time.

Provincial ‘nutrition data’ came from two main sources. One was an annual national surveillance survey on nutrition, collected by provincial Center for Preventive Medicine (CPM) under the technical support and supervision of NIN. The other was data bi-annually collected by the Center for Reproductive Health (CRH) on children’s weight and height (referred as ‘local data’). These two datasets did not always match on prevalence estimates, and there lacked consensus as to which dataset represented the actual situation in the provinces. Moreover, the final national survey data was made available six or nine months after the deadline for submitting the PPN. Consequently, provinces were not able to use the most up-to-date data but instead used the national data from the previous year. Despite these issues, the concept of SMART objectives was introduced for provincial planning, and some provinces used local data in the formulation of their plans; which data set to use appears to have been up to individual planners.

There were data discrepancies between the province and the Institute [NIN] sources, and the PCs [People's Committee] are often unsatisfied when the Institute announced the malnutrition rates when the province reported a different rate based on 6 months and 1 year data with a larger sample. (respondent from Provincial Health Service PHS)

In terms of annual ‘nutrition objectives’, the NIN provided guidance in setting targets for undernutrition and IYCF outcomes based on actual conditions of each province shown by the data, but every year the central government still assigned targets for all provinces, which were often not aligned to local realities, making it difficult for the provinces to achieve. For instance, it was reported by one respondent that PPN objectives can only be finalized once the budget needed to support the requisite activities is confirmed, but the budget is confirmed after the due date of the PPN plans; similarly another respondent reported that objectives have to be in line with national targets, even if the actual situation in a particular province for a particular issue is strikingly different to the national average. This muddled the planning process, as the evidence-based targets were often superseded by politically motivated national dictates, and funding often did not follow activities, so plans had to be revised accordingly.

**Figure 2.**
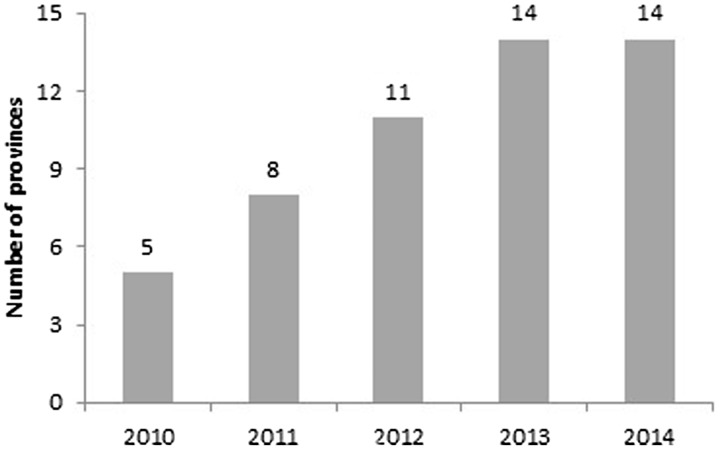
Number of provinces with objectives rated as ‘good’ according to SMART criteria from 2010 to 2014

Guidance on identifying ‘strategic solutions’ according to local data and objectives was provided by central government. Five strategic solutions are given for the provinces to select, including reduction of stunting, underweight, low birth weight and obesity. Provinces then had flexibility in listing activities under these to make them locally appropriate. Regardless of the current prevalence in the province, almost all provinces set objectives to reduce underweight and stunting. Regarding setting ‘good’ objectives according to SMART criteria from 2010 to 2014, there was an increase in the number of provinces over time, from five provinces in 2010 to all 14 available plans in 2013 and 2014 (Figure 2).

**Figure 3.**
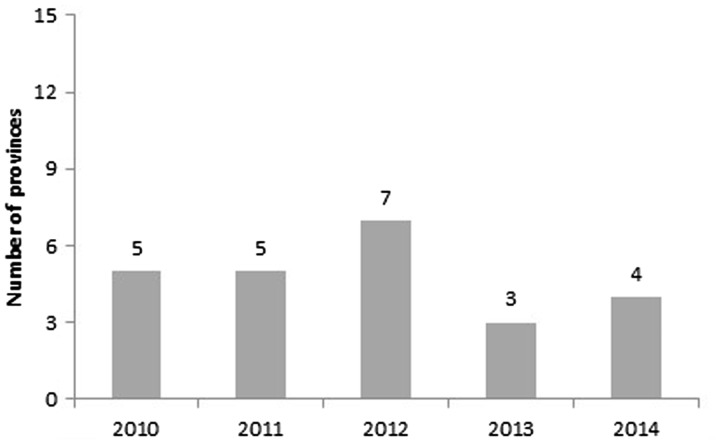
Number of provinces that include IYCF indicators to monitor nutrition objectives

IYCF is a key component of good nutrition, and a major focus of A&T. The number of provinces that ‘incorporated IYCF indicators’ as a strategy for monitoring achievements of their nutrition objectives showed an inconsistent trend over time (Figure 3). Five provinces never used IYCF indicators during the 5-year period, two provinces showed a negative change, and the remaining seven provinces did not have a consistent pattern (data not shown). The greater number of provinces using these in 2010–12 was related to the presence and push from A&T during those years. This raises sustainability challenges; however, if the PPN process and outcomes are driven so strongly by external entities.

**Figure 4.**
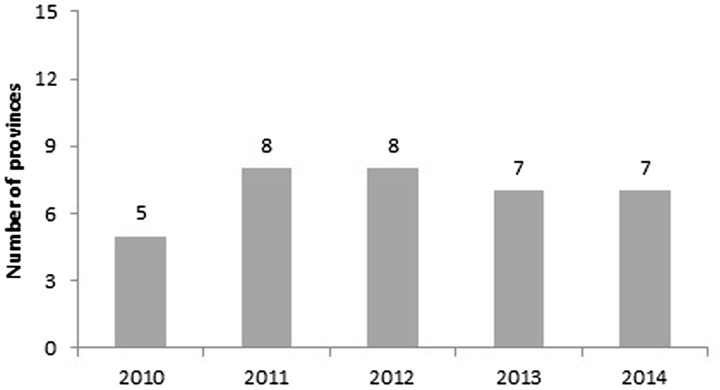
Number of provinces with multisectoral integration from 2010 to 2014

To effectively reduce malnutrition, ‘actions from multiple sectors’ are required. Since 2010 inter-agency involvement and collaboration have reportedly improved; the PHS now plays active role in mobilizing interagency cooperation with other sectors and organizations, and strengthening cooperation and partnership is one major group of solutions included in every province’s PPN. The number of PPNs that involved multiple sectors increased from 2010 (Figure 4), but in some cases ‘involvement’ included only the invitation of the partner to implement pre-specified activities, rather than active participation in the planning process. Most organizations outside the health sector reportedly considered malnutrition prevention the responsibility of the health sector and assumed the health sector receives enough resources and support to do the job. In some provinces, there was still lack of consensus between the health sector and other sectors in identifying appropriate interventions or solutions to addressing the issues. While horizontal coordination between sectors improved in some provinces, in others there remained lack of communication between the three centers that were involved in doing the planning (CRH, CPM and National Centre for Health Communication and Education (CHCE)), or with other health organizations.

In the past, under leadership of PHS, CRH and the CPM under PEMC were the only two implementing agencies involved in the development and implementation of PPN. Since 2011, PHS has invited other agencies to participate in our program planning process. (respondent from CRH)

The implementation of the activities involves the participation of different organizations, especially at lower levels. These organizations are not involved in the planning process but during the plan implementation. For example, in order to have a lot of children come for Vitamin A, we have to ask for support from the village healthcare workers, volunteers, staff of the women union, and others. (respondent from CPM)

### Issues affecting change

Both the ‘guidelines’ governing what goes into a plan and the ‘process’ of planning and review are important for provincial planning for nutrition. Support from A&T over the 4 years at both central and local level was recognized by local actors.

A&T has provided strong support in planning. They provide technical information, training on planning method, and financial support to conduct provincial workshop on PPN and carry out some communication and advocacy activities on nutrition. A&T’s support in the past 4 years is very important. (respondent from CRH)

Before 2010, NIN fixed the funding level, the list of activities, and the amount that provinces could use for each of the listed activities; plan preparation was limited to copy-and-paste of the contents from NIN’s template and plugging in guesses of prevalence numbers and assumptions from the staff in charge of planning. Based on the NIN guidelines, the PHS simply assigned targets to subordinate and implementing organizations, which reportedly caused difficulties for the provinces during implementation as the plans were not based on local context, needs, or resources.

We basically used our subjective assumptions. In other words, how we feel we should build our plan for this year. Everything is based on our feeling, including any ideas for adding or removing activities into or from the plan… Generally, we need to ensure that our plan meets our leadership’s direction, but it has to be facilitating enough for us to ensure a feasible implementation (respondent from CHEC)

…the previous guidelines were very general and lack of consideration of the specific situation in different geographic regions. Every province was asked to do the same thing, achieve the same objectives and planned targets. In addition the budget lines were fixed and basically the provinces were not allowed to make any changes (respondent from PHS)

**Figure 5.**
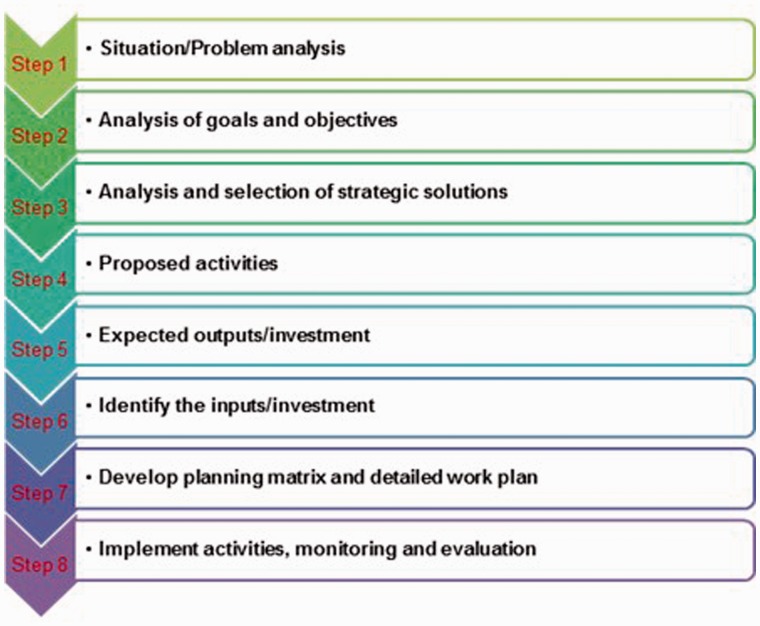
New PPN guidelines developed in 2011

After findings that the existing PPN ‘guidelines’ were overly-complex, work by A&T and others with central government and NIN resulted in streamlined PPN guidelines (Figure 5) to provide clarity on how plans should be formed, which focused on evidence- and data-based planning. The new guidelines, issued by the NIN in 2012, were reported to be more specific and realistic while still allowing some flexibility for the provinces in developing and implementing their plan.

The strong feature is that the guidelines provide details on how to develop the general and the specific objectives as well as solutions and planned targets for achieving the objectives. While it helps provinces develop a very comprehensive plan with all these elements… the feasibility of the plan is decided by the available resources from the government, including central and local government and the collaboration among different sectors (respondent from CPM)

Following the guidelines, however, was still reported to be a challenge. The guidelines were said to be not detailed enough, guidance on cost norms and pay rates were unreasonable, and they did not create room for creativity. It was a challenge for some provinces to follow the guidance due to differences between national and local priorities and objectives. Also cited was a lack of vertical coordination between institutions involved in the province and the NIN at central level, and a lack of consensus between the PHS and the plan review and approval authorities in deciding on appropriate solutions and or activities to address the local issues. For example, while the PHS saw the need to train and support community collaborators, other authorities argued that the resources could be used to purchase nutritional products to support underweight and stunted children.

One of the constraints in following the national directions is lack of flexibility in implementing the activity. For example, the national planned target is to have a radio broadcast episode in each of the commune on monthly basis… For big communes, having only 1 session on monthly basis does not make any sense but we still have to follow this direction. (respondent from CHEC)

The ‘process’ of provincial planning was identified in baseline research as too complex and top-down, and therefore an impediment to effective decentralized planning. The planning process includes a workshop conducted by NIN to discuss the guidelines for PPN and nationally assigned targets, solutions, and funding levels for each of the provinces; drafting of the plan by the three primary stakeholders in each province (CRH, CHEC and CPM) based on the role, function and scope of work of each center; and plan review and approval by several national bodies, including a decision on allocating resources for implementation and incorporating the plan into the provincial socioeconomic development plan.

Although decentralization was said to be important and the PPN guidelines emphasized context in the content of the plans, the planning process itself appears to have changed little over time, with planning still seen as a top-down exercise focused on allocating financial resources. The review and approval process was reported to be too long, with reviewers only paying attention to the general guidance rather than considering the actual situation of the province. There is, therefore, reportedly, a mismatch between the planning process and the content of the plans set in the guidance.

I have not seen any changes in how the plan is developed in the past 4 years. It is developed based on the objectives and planned targets set by the National Institute of Nutrition. There have been no changes in the way we do our planning. If there are any changes to the NIN’s plan then we make changes to our plan accordingly (respondent from CHEC)

‘Leadership’ and staff ‘capacity’ were commonly reported constraints. The leadership role of the PHS in provincial planning on nutrition has not always been clear and decisive. The PHS did not seem to have a well-defined strategy for nutrition programs, and its activities were apparently limited to reviewing and combining the contents of the plans submitted by the three centers (CRH, CPM and CHEC). Although staff knowledge and ability improved over time, there was insufficient staff and the staff in place did not have sufficient planning capacity. Due to these constraints, local planning staff became dependent on the national guidelines, stifling creativity or local relevance. Similarly, plan reviewing activities in the Provinces were limited to ensuring compliance with legal documents (guidance and regulations) rather than conducting a real needs assessment to identify what could be done better to support the implementation of the PPN.

They [staff] know how to define the local objectives as compared to the national objectives based on local conditions, including human resource, funding, geographic coverage, etc. They know how to do the review of available budget and resources, including funding from national target programs, from local government and from projects supported by NGO (respondent from CHEC)

Yet, we have more issues with planning at district level. This is because there is no full time staff in charge of PPN planning and implementation at district level. Staff turnover makes it difficult for our training to be effective. We find it challenging in providing guidance on program planning to staff at district level. Though we have provided due guidance, reporting [back to Province] seems to be a pending issue (respondent from CRH)

To implement plans, ‘resource mobilization’ needs to be adequate. Funds for implementing, the PPN comes from the national government as the primary source, and from the local government and potentially other local sources (NGOs and business) as supplementary. Both total funds from all sources and central funding as a proportion of total funds have declined for nutrition in all provinces except one (of 12 with data available) since 2010, and the approved budget for implementing the PPN was usually (though not always) lower than the budget level needed. National funding is generally tied to pre-defined activities, and so does not always match with decentralized plans even after efforts to improve decentralized planning for nutrition, and this mismatch remains a major constraint to setting context-specific targets and activities. In the past three years, the national budget for nutrition has been decreased by 40–80% while the objectives and planned targets in NIN’s guidelines were usually not adjusted; similarly, although improvement of nutrition status was included in the provincial socioeconomic development agenda, the local government’s investments in nutrition activities were insufficient in most provinces. Some provinces were collecting (or considering) a fee for using the nutrition and IYCF counseling services at healthcare facilities, which could potentially limit access, and others had had to set priorities and identify core activities. Planning and budgeting was generally an iterative process, with plans being written according to need, then revised according to available resources and national requirements. A&T support in mobilizing funds from local stakeholders was reported as useful by some respondents, but is not a sustainable solution.

The challenge in doing the planning is that we can only finalize the plan when information on approved budget is available. We never know about the certainty of funding for individual activity for the next year in order to do appropriate planning. For example for we include in our plan a training activity but the funding for training might not be available in the approved budget. Likewise the approved funding for the nutrition survey might not match the proposed funding for the activity then we have to do lots of revisions to the final plan. (respondent from CPM)

### Summary

Despite this mixed picture from analysis of the content of PPNs and the process of making them, two-thirds of stakeholders reported that the plans had improved. This was partly attributed to A&T workshops and training, which improved capacity and availability of data. Respondents did cite, however, significant limitations in applying new learning and systems from remnants of centralized planning processes; if activities or targets outside of the annual national nutrition plans and multi-year national plans of action on nutrition were included, the PPN was usually rejected by the PHS or other authorities and was redone. In addition, there was conflict between documents and regulations issued by the central government. A&T did work with national government to develop guidance for the PPN process, but these were still constrained by centrally planned national priorities and targets on which A&T had no input.

The A&T training is very comprehensive and in-depth. I was able to develop more understanding about some technical issues but also on planning process. Their training is very useful. The training is very good and very practical. Unfortunately it is not applicable in reality. We still have to follow the national framework in doing the planning. (respondent from CPM)

If we develop the plan based on our actual needs and independently from the availability of the national and local funding, our proposed budget would never be approved. This also makes our annual planning process just a formality…If it was not following the guidelines, the PPN would not get approved by either the PHS or other authorities such as the People’s Committee or the DoF [Department of Finance]. It would be very difficult to get it approved. (respondent from CHEC)

In terms of policy or legal environment, there are some challenges encountered during the implementation. For example, the plan might have been developed based on guidance from a circular or an ordinance but later on an official document was sent out saying that the circular or the ordinance was no longer applicable. This creates confusions for the planning staff… There are overlapping and also conflicts between different policies. (respondent from PHS)

I think if changes are made to the PPN process, it must start with the NIN. The NIN should provide more specific guidance for the planning, including what activities are prioritized and for what target groups. This is because the PPN is based on the national guidance or guidelines from the NIN and must fit in the National Nutrition Strategy. It is also important to provide direction on funding allocation for implementing the national strategy or action plan. (respondent from CPM)

This assessment of changes in Provincial Planning for Nutrition has uncovered important evidence on the use of data, clear objectives, strategic solutions and IYCF indicators in plans; assessed multi-sector action, leadership, and capacity in the process of bringing plans together; considered the roles of revised guidelines, resources, and the regulatory environment in creating and implementing plans; and reflected on the role of A&T in changes that occurred. Although the picture is complex and results therefore mixed, we are able to draw some lessons going forward.

## Discussion

Research undertaken before the A&T program began in 2009 called for stronger engagement of provinces in nutrition planning, as these are the center of decision making and resource allocation under the partial decentralization of the health sector in Vietnam (Lapping *et al.* 2014). This 2014 paper noted several potential constraints to this action—including continued centralized control, and limited resources, data, capacity and collaboration—which A&T explicitly set out to address.

Our evaluation of the PPN process, drawing on stakeholder interviews in 2010 and 2014 and annual PPN content analysis highlights improvements in the quality of nutrition plans but also significant continued planning challenges. The number of provinces with broadly adequate or good nutrition plans rose over time, and some elements in the plans grew stronger. Local planning skills improved, but capacity remained insufficient. Awareness of and support for nutrition improved, but some policy and legal environments were contradictory. Objectives were clearer, but use of data for planning remained inconsistent. Provinces became more proactive and creative, but remained constrained by slow central approval processes and insufficient funding. This suggests that improvements occurred, particularly at the provincial level where A&T work was focused, but also suggests that there are continued challenges, particularly from the national level where more work might be done to relieve constraints on planning. The broader politics, however, will likely remain a limiting issue.

The contents of PPNs improved between 2010 and 2014. Some provinces applied local nutrition data to the development of their PPNs, but a similar number of provinces did not; the facilitating role played by A&T in making available local data was helpful, but unless this role is institutionalized for the long term, it is not a sustainable solution. Although nutrition objectives were well-stated by most provinces, these continued to be influenced by central-level requirements. Involving other sectors in a meaningful way presents a real challenge; planners may recognize the importance of involving sectors other than health, but often stop at mentioning the sectors in the plan without any details about their exact involvement. Lack of consensus and collaboration among concerned organizations, and absence of some critical topics in the PPN guidelines, continued to be reported as major challenges.

Issues with the process of decentralized planning hampered these efforts. Capacity was cited as a major constraint by respondents; although capacity improved, there remained lack of confidence for undertaking the planning process. This is echoed in other assessments of public service decentralization in Vietnam (Wit 2007), where it was found that three quarters of local staff members do not have proper training for their role, and elsewhere (Azfar *et al.* 1999). This lack of capacity throughout the Vietnamese health system has been attributed to disruption dating back to economic reforms in the 1980s, which undermined funding for training and health service use (Lieberman *et al.* 2005). Even with improved guidelines then, it could take more years of capacity development and support to improve planning processes in a sustained manner, even if central institutions ease their influence over planning.

Administrative decentralization is advanced in Vietnam, but for public services other than nutrition it has been noted that, while tasks and funds have been allocated to provinces, power and decision-making has remained centralized (Lieberman *et al.* 2005; Wit 2007). The same appears to be true for nutrition; while the rationale and plan for decentralized nutrition planning is sound, the central level needs to catch up with the provinces. The main proposed advantages of decentralization will not be achieved consistently or in a meaningful way if decentralized nutrition planning remains constrained by relatively inflexible centralized planning processes.

Fiscal decentralization has been applied sporadically and by default in the PPN process, with funds still tied to national objectives rather than decentralized ones. For many years, provinces have been dependent on the availability of national funding, but central government has been withdrawing funds over time. Furthermore, after 2015 this national funding was cut by 65% due to reductions in donor funds stemming from to Vietnam’s graduation to ‘middle-income country’ status in 2008; if national government does not see nutrition as a priority in this new funding situation, provinces will become more challenged by the lack of resources necessary for the implementation of the PPN. On the one hand, this might be seen as an opportunity for provinces to make better efforts in mobilizing resources from different sources and having more autonomy in planning, if the review and approval process allows this. On the other, it has been suggested in other Asian contexts that decentralized nutrition programming can suffer from reduced economies of scale (Friedman *et al.* 2006). A&T’s support in resource mobilization played an important part in helping provinces think ahead about the sustainability of PPN, but this should be within a broader debate about fiscal decentralization in Vietnam, and whether it would truly be allowed in practice.

The major narrative coming from this research is that, despite positive work to support planning systems and raise awareness and capacity, critical constraints remain at provincial level—many of which are outside the control of external projects trying to bring change, at least in the short term, and all of which chime with the few prior studies of nutrition decentralization in Asia in general (Friedman *et al.* 2006) and health system decentralization in Vietnam in particular (Lieberman *et al.* 2005). What changes there have been at provincial level have been constrained primarily by the maintenance of top-down centralized planning, budgeting, and review processes, which inhibit the contextualization of provincial plans and creativity in local programming. Vietnam’s decentralization more broadly has been found to be constrained by a lack of incentives for bureaucratic actors to transfer power and resources to local levels, and ill-defined boundaries between functions controlled by central-level managers and those controlled by their field level counterparts (Omar 2002). If incentives and boundaries cannot be defined and acted upon, all of the local-level capacity building and formulating of clear and rational goals that were supported by A&T likely will not be able to achieve long-term and comprehensive decentralized nutrition planning. In other contexts, what works best is often a practical hybrid of authoritative (often central) coordination, local problem solving, and borrowing from local ways of working in particular contexts (Crook and Booth 2011), with the role of the center more one of redistributing resources and providing technical assistance (Fritzen 2006). This may be a useful solution for moving provincial nutrition planning in Vietnam forward given the preferences currently for maintenance of centralized power and given the existing nutrition situation in Vietnam. Although the nutrition situation has improved substantially over recent years in Vietnam, future improvements in nutrition will require targeted attention to behaviors in sub-groups that must be tailored to local contexts to be effective, given the substantial ethnic, socioeconomic, and ecological diversity in the country. Working even this hybrid solution; however, will require greater clarity on boundaries and roles of actors at different levels; previous work has also found that, in addition to improved technical ability, local administrators require clear information on expectations for the decentralization process itself (Azfar *et al.* 2001; Wit 2007).

This study is one of few globally that has followed the process of decentralization of nutrition planning which is occurring in many different contexts, and attempted to understand and improve the process. Although this research has some important results which resonate with other published literature on nutrition and health system decentralization in Vietnam, the study has some limitations. Most importantly, the design of the project did not allow us to compare findings in the A&T Provinces with other, unsupported Provinces, and we do not therefore have a counterfactual to assess what was happening in the absence of the program. We have provided, however, a detailed assessment of change in those Provinces with A&T support. Second, we may not have covered all possible respondents and therefore all possible viewpoints. Through having a team based in Vietnam for the duration of the project; however, we had a good overview of those at national and provincial level who were involved in the PPN process, and are confident that the most important viewpoints have been captured. Finally, the complex and multi-faceted nature of the plans, the process, and the context necessitated some simplification and ordering in order to undertake this assessment; we believe that the trade-off between synthesis and complexity was balanced in our study.

In conclusion, targeted assistance and local advocacy can improve some components of decentralized planning for nutrition, with success dependent on the overall policy and programming context and ability to overcome constraints around capacity, investment, and remnants of highly centralized planning. We recommend strong engagement with planners at the national level to understand how to unblock major constraints; solutions must take into consideration the particular political, financial and administrative context.

## Ethical approval

The study received Institutional Review Board approval from the International Food Policy Research Institute (IFPRI) in the USA and from the Center for Creative Initiatives in Health and Population in Vietnam. NIN and the Center for Reproductive Health (CRH) in each of the participating provinces gave verbal consent to use the PPNs for content analysis. Verbal consent for participating and having the interviews tape-recorded were obtained for all interview participants, and the endline interview research protocol was reviewed and approved by the Institute of Social and Medical Studies in Vietnam.

## Notes

^1^Note that not all 15 provinces provided a full plan in each year, hence numbers not adding up to 15 in all years.
